# Discriminative and Quantitative Analysis of Antineoplastic Taxane Drugs Using a Handheld Raman Spectrometer

**DOI:** 10.1155/2018/8746729

**Published:** 2018-07-03

**Authors:** Laetitia Lê, Marion Berge, Ali Tfayli, Patrice Prognon, Eric Caudron

**Affiliations:** ^1^U-Psud, Univ. Paris-Saclay, Lip(Sys)^2^, EA7357, UFR-Pharmacy, Châtenay-Malabry, France; ^2^European Georges Pompidou Hospital (AP-HP), Pharmacy Department, Paris, France

## Abstract

This study was conducted to evaluate the ability of Raman spectroscopy (RS) to control antineoplastic preparations used for chemotherapy in order to ensure its physical and chemical qualities. Three taxane drugs: cabazitaxel (CBX), docetaxel (DCX) and paclitaxel (PCX) at therapeutic concentration ranges were analyzed using a handheld spectrometer at 785 nm. Qualitative and quantitative models were developed and optimized using a calibration set (n=75 per drug) by partial least square discriminant analysis and regression and validated using a test set (n=27 per drug). All samples were correctly assigned with an accuracy of 100%. Despite optimization, quantitative analysis showed limited performances at the lowest concentrations. The root mean square error of predictions ranged from 0.012 mg/mL for CBX to 0.048 mg/mL for DCX with a minimal coefficient of determination of 0.9598. The linearity range was validated from 0.175 to 0.30 mg/mL for CBX, from 0.40 to 1.00 mg/mL for DCX and from 0.57 to 1.20 mg/mL for PCX. Despite some limitations, this study confirms the potential of RS to control these drugs and also provides substantial advantages to secure the activity for healthcare workers. As a result of its rapidity and the uncomplicated use of a handheld instrument, RS appears to be a promising method to augment security of the medication preparation process in hospitals.

## 1. Introduction

A number of strategies including surgery, radiotherapy, and systemic therapy can be used and combined to treat cancer patients. Chemotherapy using antineoplastic drugs is one of the major strategies; nowadays more than a hundred drugs are used. They are used alone or in combination with other drugs or treatments to optimize the medication process. Despite different chemical compositions or mechanisms, antineoplastic drugs act over all by stopping or slowing the growth of cancer cells.

In most cases, intravenous antineoplastic drugs are prepared just before use by pharmacy technicians in aseptic conditions in a centralized hospital pharmacy. Drugs are diluted in 0.9% sodium chloride or 5% glucose solution to obtain the concentration prescribed for the patient by physician.

In order to ensure the physical and chemical quality of the preparation, numerous quality control strategies have been developed to ensure that the right drug is delivered at the right concentration [1]. High performance liquid chromatography with UV detection (HPLC/UV) was developed in our hospital and is one of the oldest approaches for the control of these drugs [2–6]. Recently, new technologies have combined near-infrared and UV spectroscopies (NIR-UV) or Raman spectroscopy with UV detection (Raman-UV) that are tending to become among the most widely used analytical techniques [7–11]. These techniques have been used to control numerous antineoplastic drugs and also monoclonal antibodies; qualitative analyses are conducted on UV spectral data and extended by NIR or Raman spectra when UV data are not sufficiently discriminant. Quantitative analyses, however, have been reported only for statistical analysis on UV data, explaining the comparative performances observed with flow injection analysis (FIA) or HPLC/UV.

For several years, NIR and Raman spectroscopies have each been described as noninvasive, nondestructive, and rapid methods to analyze drugs [9, 12–15]. Resulting from the considerable absorption of water in the NIR region, Raman spectroscopy is preferred for the analysis of molecule in an aqueous environment. This technique is now recognized by the US Pharmacopeia as one of the accepted technologies for material identification and verification [16]. Raman spectroscopy is a vibrational molecular technique, providing data on vibrations of molecular bonds, such as stretching or bending, by interaction of an incident beam of monochromatic light (*υ*_0_) with molecules in the sample. Raman scattering occurs when the electric field of the excitation light induces a change in the polarizability of the bond and consequently the emission of two types of Raman photons: Stokes (*υ*_0_ − *υ*_v_ with *υ*_v_ corresponding to the vibration frequency of the molecular bond) and anti-Stokes (*υ*_0_ + *υ*_v_) observed in Raman spectra. Raman spectra therefore provide information on the nature and structure of molecules in the sample and also their interactions with the environment [17]. This technique is now widely used in the pharmaceutical industry to identify raw materials before production, falsified medicines and also in the process analytical technology (PAT) [18, 19]. Regarding PAT, the control approach of process should be at-line, on-line, or in-line in order to take into account the increasing difficulty of the implementation of process analyzers.

Regarding the risk of toxicity of antineoplastic drugs, Raman spectroscopy presents substantial advantages for the safe analytical control of antineoplastic drugs. At the present time in hospital, the standard analytical procedures for controlling cytotoxic preparations based primarily on the UV spectral characteristic of drugs are invasive [2]. These techniques require a sample of the preparation that may expose healthcare workers to risk, for example, pharmacists who prepare and control antineoplastic drugs and nurses who administer them [20–22]. Compared to common techniques involving UV properties, Raman spectroscopy enables noninvasive and nondestructive analysis, and measurements can be done through transparent materials such as plastic or glass containers [11, 12]. Regarding the inherent toxicity of these drugs, especially in terms of carcinogenic, mutagenic, and teratogenic properties, noninvasive measurements are particularly interesting for limiting contacts between operators and drugs and thereby limiting occupational exposure. Nevertheless, despite proportionality of Raman shift and concentration of chemical components, Raman spectra are very complex data difficult to interpret. Specific multivariate analysis developments are consequently required to facilitate the access to the relevant information or to extract the information sought. Despite rapid analysis, nondestructive measurement and the ability to conduct analysis* in situ*, these difficulties explained the slow development of Raman spectroscopy in hospital pharmacies.

The aim of this study was to confirm the ability of Raman spectroscopy to control antineoplastic drugs at different concentrations. In order to evaluate the limitations of this technique, the study focused on three drugs, very often prepared in our hospital and among the most critical ones to discriminate as a result of similar chemical structures and low concentrations.

## 2. Material and Methods

### 2.1. Drugs

**Figure 1:**
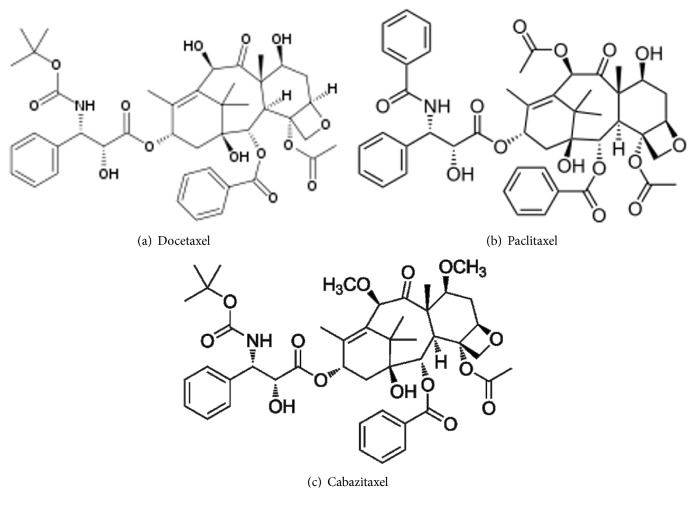
Molecular structure of the three taxane antineoplastic drugs: docetaxel (a), paclitaxel (b), and cabazitaxel (c).

Taxane drugs are among the most effective and commonly used systemic therapies for breast cancers, particularly in the adjuvant context. Taxanes bind to beta-tubulins, promoting the assembly of microtubules, inhibiting depolymerization and therefore the cell division process. At the present time, three taxanes (Figure 1) have been approved by the FDA and several combination therapies or single analogue drugs are currently in development or are the subject of clinical trials. All approved formulations are intravenously administered.

Commercial aqueous formulations of docetaxel (DCX), paclitaxel (PCX), and cabazitaxel (CBX) were obtained from Hospira®, Kabi®, and Sanofi®, respectively. Taxanes are poorly soluble in aqueous solution because of their bulky and fused-ring skeleton with lipophilic substituents. Specific excipients are therefore required to solubilize or stabilize these drugs in formulations such as Cremophor EL (polyethylated castor oil: CrEL) and polysorbate 80 (Tween 80), which can entrap taxanes in water by forming micelles, or citric acid in cabazitaxel formulations to adjust the pH and stabilize the molecule in solution. DCX is marketed at 10 mg/mL with anhydrous ethanol, citric acid, Macrogol 300, and polysorbate 80 as excipients. PCX was formulated at 6 mg/mL with anhydrous ethanol and macrogolglycerol ricinoleate. Sixty g of CBX was lyophilized, yielding a concentration of 40 mg/mL after reconstitution with citric acid, anhydrous ethanol, and polysorbate 80.

### 2.2. Sample Preparation

There exist no specific guidelines for controlling drugs by Raman spectroscopy, and so samples were prepared according to the guidelines for use of near-infrared spectroscopy published by the European Medicines Agency [23].

Five series of solutions of each drug were prepared in aseptic conditions by dilution in 0.9% sodium chloride solution (FreeFlex® isotonic saline, Fresenus-Kabi, France: a different batch of sodium chloride solution per series to account for variability) at various concentrations which covered the entire therapeutic range. Each solution was packaged in glass vials (Interchim®, Montluçon, France) for analysis.

### 2.3. Instrumentation: Handheld Raman Spectroscopy

Metrohm Instant Raman Analyzer (MIRA, Metrohm, France) was used to obtain Raman spectral acquisitions. The excitation source was a 785 nm single-mode diode laser generating a maximum of 75 mW on the sample. Analyses were conducted using the vial module at a focal distance of 1.0 mm with the orbital raster scan (ORS) system. The use of ORS technology allows analyzing a larger volume of the sample which leads to increase in the accuracy, repeatability, and reliability of the measurements. The spectral region studied was 400–2300 cm^−1^ with a spectral resolution from 12 to 14 cm^−1^ and acquisition time was 8 seconds. Spectral acquisition and data preprocessing were conducted with Metrohm Mira software (Metrohm, France). Samples were analyzed in glass vials in triplicate in order to include container-induced variability.

### 2.4. Chemometric Analysis

Raman spectra were normalized over the entire spectral area from 400 to 2300 cm^−1^. All data were analyzed with a principal component analysis (PCA) to highlight and exclude aberrant spectra. MATLAB® 7.12.0 (R2011a) software was used for chemometric analyses.

Calibration models for qualitative and quantitative analyses were developed, optimized, and validated according to Guidelines of the Use of NIRS by the Pharmaceutical Industry published by the European Medicines Agency [23]. Three sets were constituted: (i) a calibration set for creating the calibration model, (ii) a calibration test set for internal validation and optimization of the model, and (iii) an independent validation set for external validation of the model. According to EMEA guidelines, the calibration test set was grouped to the calibration set to increase the variability in the calibration set, thus the robustness of the models. Consequently, a k-fold cross-validation technique (k = 20) was applied to develop and optimize predictive models.

For each drug, 75 spectra (5 series of experiments, 5 concentration levels per series, and 3 replicates per concentration levels and per series) were included in the calibration set. Concentrations varied from 0.05 to 0.25 mg/mL for CBX, from 0.20 to 0.75 mg/mL for DCX, and 0.24 to 1.20 mg/mL for PCX. In addition, 27 new spectra (3 series of experiments, 3 concentration levels per series, and 3 replicates per concentration levels and per series) were prepared for each drug and used for the validation set: at 0.12, 0.18, and 0.25 mg/mL for CBX, at 0.40, 0.60, and 0.80 mg/mL for DCX, and at 0.42, 0.60, and 0.97 mg/mL for PCX.

In order to limit noninformative spectral background, various spectral processing methods were examined, including first and second Savitzky-Golay derivatives (4th-order polynomial; width: 7 points), baseline correction, standard normal variate (SNV), and combined pretreatments.

Qualitative analysis involved a partial least square discriminant analysis (PLS-DA) to differentiate samples of the three taxane drugs. As a standard tool of chemometric analysis, PLS-DA involves a supervised classification method based on classical PLS regression. According to this method, the number of original variables is reduced in order to retain only the relevant characteristics of spectra that most contributed to its variance. For each model, several data scaling methods (autoscaling, mean centering) and Bayesian PLS-DA were evaluated manually using the classification toolbox. The optimal number of components was considered for the lowest error rate of fitting and cross-validation in classification by 10-fold cross-validation. The best predictive models were selected for the lowest cross-validation error, the lowest calibration error, and the minimal number of unassigned samples.

Quantitative analysis involved PLS regression. For both qualitative and quantitative analyses, the number of latent variables of each model tested was previously optimized by leave-one-out-cross-validation. The predictive capacity of regression models was assessed by the root mean square error of cross-validation (RMSECV) calculated from the calibration set and the root mean square error of prediction (RMSEP) was calculated from the external validation set. These errors are derived from the RMSE formula, where y_i_ is the theoretical concentration of sample, $\hat{y_{i}}$ is the predicted concentration, and n is the total number of samples. The optimal predictive model was selected for the lowest RMSECV and RMSEP errors with the highest coefficient of determination (R^2^).

(1) $$\begin{array}{c} RMSE=\sqrt{\frac{1}{n}\overset{n}{\underset{i=1}{\sum }}{\left(y_{i}-\hat{y_{i}}\right)}^{2}}. \end{array}$$

Calibration models were then validated in accordance with ICH Q2 R1 and SFSTP (French Society of Pharmaceutical Sciences and Techniques) guidelines [24–26]. To assess the validity of the model, an approach based on accuracy profiles was performed. Tolerance intervals were calculated for the predicted concentration of the validation set with a *β*-expectation tolerance of 90% and compared to acceptance limits set fixed at ± 15% for all concentrations.

## 3. Results and Discussion

**Figure 2:**
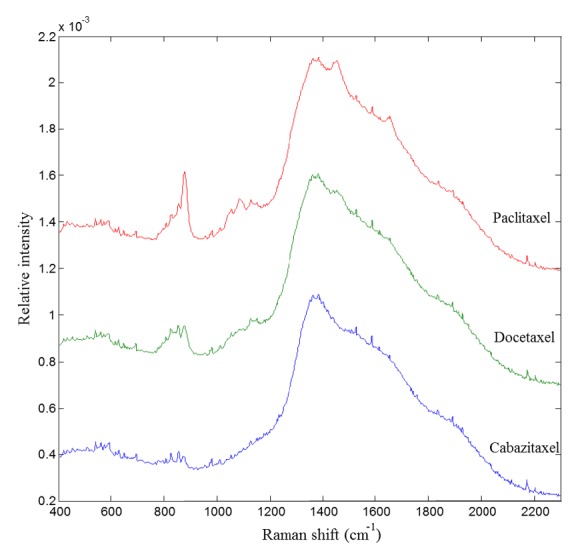
Mean Raman spectra of cabazitaxel (from 0.05 to 0.25 mg/mL), docetaxel (from 0.20 to 0.75 mg/mL), and paclitaxel (from 0.24 to 1.20 mg/mL) diluted in 0.9% sodium chloride solution normalized over the total spectral range from 400 to 2300 cm^−1^.

Average Raman spectra from 400 to 2300 cm^−1^ are shown in Figure 2.

### 3.1. Discriminant Analysis

**Figure 3:**
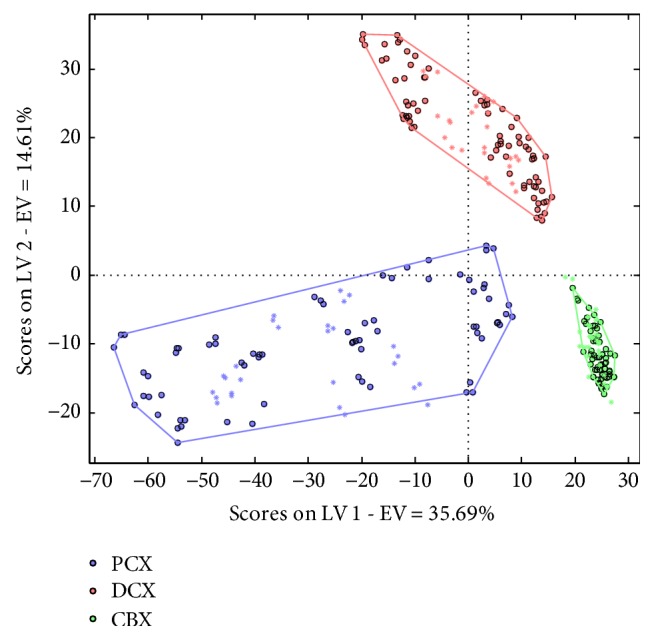
Scatterplot scores of Raman spectra for the PLS-DA model developed from Raman spectral data preprocessed by Der1 and SNV. Samples of the training set are represented by a circle and samples of the external validation set by an asterisk.

The PLS-DA multivariate statistical technique was used to discriminate the three taxane drugs in chemotherapy preparations. The best PLS-DA was obtained for Raman spectral data pretreated by Der1 and SNV. Five significant latent variables (LVs) were selected to develop the classification model that corresponds to 64% of the total variability of the original variables (35.69% associated with the first LV, 14.61% with the second variable, and 13.7% explained by the other three LVs).  Figure 3 shows the scatterplot scores of Raman spectra for the two first LVs and illustrates the good clustering of CBX, DCX, and PCX samples. In addition and to discriminate DCX from PCX samples, the first LV also reflects the influence of the concentration of PACX, characterized by a large dispersion of samples of the calibration and validation sets. CBX samples, however, were discriminated from other classes primarily according to the second LV. According to this model, all samples were correctly assigned with an accuracy of 100%.

### 3.2. Quantitative Analysis

**Figure 4:**
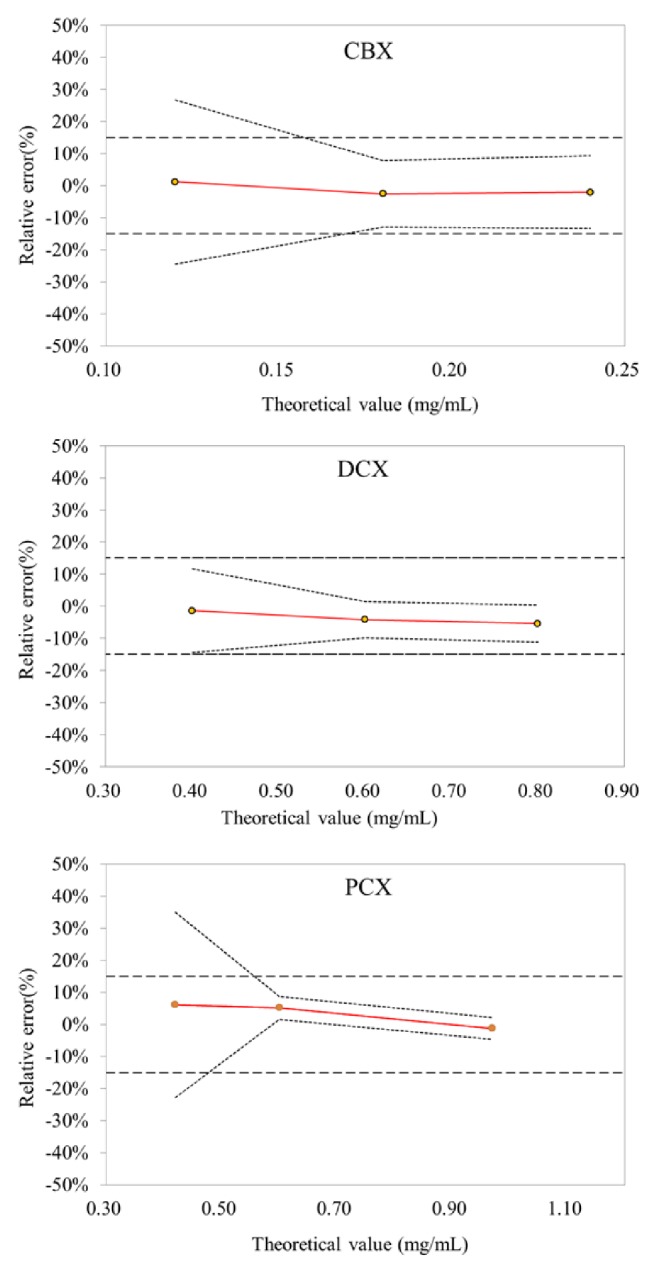
Accuracy profiles obtained for the validation of taxane drugs quantification in sodium chloride solution by considering linear regression. The dashed lines are the upper and lower acceptance limits set at 15%, the dotted lines are the upper and lower *β*-expectation tolerance limits (with *β* = 90%), and the red solid line is the relative bias.

In order to optimize the calibration models, numerous preprocessings were assessed and used to develop PLS regression models. The best predictive models were selected for the lowest root mean square error of prediction (RMSEP), the lowest root mean square error of cross-validation (RMSECV), and the maximal coefficient of determination (R^2^). Based on the predicted concentration values obtained for samples of the validation set, the accuracy profiles and the ICH Q2 criteria of validation were calculated for each drug (Figure 4). The lower limit of quantification (LLOQ) was determined at the intercept between the tolerance interval and the acceptance limits. The performances and validation results of the selected models for each drug were presented in Tables 1 and 2.

Despite optimization, the upper and the lower *β*-expectation tolerance limits (%) exceed the acceptance limits settled to 15.0% for the lowest concentration levels. These methods are not able to provide accurate results over all the therapeutic concentration ranges. Consequently, the linearity range was limited for CBX from 0.175 to 0.3 mg/mL, for DCX from 0.40 to 1.00 mg/mL, and for PCX from 0.57 to 1.20 mg/mL.

Trueness values expressed by the relative bias (%) at each concentration level were between −5.3% and 5.1% for concentration levels included in the validated ranges. In order to assess the precision of the methods, the repeatability and intermediate precision expressed by the relative standard deviation (RSD) of the intraday standard deviation and between-day standard deviation, respectively, were also calculated. Over the validated ranges of concentration, RSD values below 6.0% were obtained for all drugs.

This study confirms the qualitative ability of Raman spectroscopy to identify and verify cytotoxic drug preparations. Total discrimination was observed over the entire therapeutic concentration range, even for low concentrations, and no sample was unassigned or misclassified. In light of these results, Raman spectroscopy is an excellent tool for guaranteeing the nature of the drug before administration to patients. Some limitations were nevertheless observed concerning quantification. Despite optimization, quantitative analyses highlighted limited sensitivity for low concentrations. For other concentrations, excellent analytical performances have been observed.

In addition to analytical performances, the small size, the robustness of the instrument, and its ease to use constitute favorable arguments for the implementation of handheld Raman analyzer. For several years, more handheld Raman analyzers have been on the market and have facilitated the use of this technology. These instruments are nevertheless generally only described for qualitative analysis and, at present, no specific library is yet available to specifically control antineoplastic drugs. Moreover, despite interesting noninvasive possibility of analysis by direct measurement through the transparent container such as glass of plastic material, specific accessories have to be developed to secure the analysis in terms of repeatability and reproducibility. Regarding promised results, it will be interesting to extend this study to other drugs and containers used to administrate treatment (diffusor, syringe, and bag) to consolidate the safety management in medication process.

## 4. Conclusion

The primary objective of this study was to develop and validate qualitative and quantitative predictive models for the analysis of antineoplastic drugs from the same therapeutic family with high similar chemical structures. Despite some limitations, this study confirms the potential of Raman spectroscopy to control these drugs. Taking into account the necessity of developing specific libraries and integrating quantitative algorithms to determine the concentration of the chemical molecule in the sample, the rapidity of this approach and the uncomplicated use of handheld instruments make Raman spectroscopy a promising alternative or complementary method to increase the safety of the medication preparation process in hospitals.

## Figures and Tables

**Table 1:** Performance of Raman calibration models for quantification developed for the entire concentration ranges. This table shows the characteristics of the calibration models with the number of spectra used to develop and validate models (n) and the number of latent variables (nLVs) considered when developing the calibration model. This table also lists the results of predictive performances including the coefficient of determination (R^2^), root mean square of cross-validation (RMSECV), and the root mean square error of prediction (RMSEP) for calibration models.

	CBX	PCX	DCX
Concentration range investigated (mg/mL)	0.05–0.30	0.24–1.20	0.2–1.00
Data pre-processing	Raw	Raw	SNV

nLVs	12	4	6
RMSECV (mg/mL)	0.014	0.084	0.055
RMSEP (mg/mL)	0.012	0.041	0.048
R^2^	0.9999	0.9598	0.9916

**Table 2:** Results coming from the validation of the Raman method developed to quantify taxane drugs in sodium chloride solution.

	CBX	PCX	DCX
Range of linearity (mg/mL)	0.175 – 0.30	0.57 – 1.20	0.40 – 1.00
Slope	1.0001	0.9598	0.9917
Intercept	7.10^−6^	0.0252	0.0048
R^2^	0.9999	0.9598	0.9909
LOQ (mg/mL)	0.16	0.56	0.40

Relative bias (%)			
Level 1	0.3	6.1	−1.4
Level 2	−2.2	5.1	−4.2
Level 3	−0.2	−1.2	−5.3
Repeatability (RSD %)			
Level 1	7.6	2.6	3.2
Level 2	2.5	1.7	2.3
Level 3	5.4	1.7	1.6
Intermediate precision (RSD %)			
Level 1	10.7	8.6	5.2
Level 2	3.9	1.8	2.7
Level 3	5.4	1.8	2.5
*β*-Expectation tolerance limit (%)			
Level 1	[−24.7;25.3]	[−22.8; 35.0]	[14.5; 11.7]
Level 2	[−11.6; 7.2]	[1.5; 8.8]	[−9.8; 1.5]
Level 3	[−10.8; 10.4]	[−4.7; 2.2]	[−11.1; 0.4]

## Data Availability

The data used to support the findings of this study are available from the corresponding author upon request.
